# Acoustic properties of vowel production in Mandarin-speaking patients with post-stroke dysarthria

**DOI:** 10.1038/s41598-018-32429-8

**Published:** 2018-09-21

**Authors:** Zhiwei Mou, Zhuoming Chen, Jing Yang, Li Xu

**Affiliations:** 1 Department of Rehabilitation, the First Affiliated Hospital of Jinan University, Guangzhou, Guangdong, 510630 China; 2 Department of Communication Sciences and Disorders, University of Wisconsin-Milwaukee, Milwaukee, WI 53211 USA; 3 School of Rehabilitation and Communication Sciences, Ohio University, Athens, Ohio, 45701 USA

## Abstract

This study investigated the acoustic features of vowel production in Mandarin-speaking patients with post-stroke dysarthria (PSD). The subjects included 31 native Mandarin-speaking patients with PSD (age: 25–83 years old) and 38 neurologically normal adults in a similar age range (age: 21–76 years old). Each subject was recorded producing a list of Mandarin monosyllables that included six monophthong vowels (i.e., /a, i, u, ɤ, y, o/) embedded in the /CV/ context. The patients’ speech samples were evaluated by two native Mandarin speakers. The evaluation scores were then used to classify all patients into two levels of severity: mild or moderate-to-severe. Formants (F1 and F2) were extracted from each vowel token. Results showed that all vowel categories in the patients with PSD were produced with more variability than in the healthy speakers. Great overlaps between vowel categories and reduced vowel space were observed in the patients. The magnitude of the vowel dispersion and overlap between vowel categories increased as a function of the severity of the disorder. The deviations of the vowel acoustic features in the patients in comparison to the healthy speakers may provide guidance for clinical rehabilitation to improve the speech intelligibility of patients with PSD.

## Introduction

Dysarthria is a class of motor speech disorders resulting from neurological injuries that causes impaired or uncoordinated movement of the muscles, including the lips, tongue, lower jaw, velum, vocal folds, and diaphragm during speech production. Compared to healthy speakers, dysarthric speakers showed reduced and centralized articulatory movement space in the vocal tract such as small tongue movement, decreasing jaw and lower lips movement^1–3^, slow pace of articulatory movement^4^, and discoordination between the articulatory gestures^5^. These articulatory deviations are commonly reflected in the speech acoustics which also result in perceptually identifiable features. These features may detrimentally affect the intelligibility of people with dysarthria. Clinically, dysarthria is categorized into seven subtypes: ataxic, flaccid, spastic, hypokinetic, hyperkinetic, unilateral upper motor neuron, and mixed. Dysarthria can be caused by various types of diseases such as stroke, Parkinson’s disease, cerebral palsy, brain trauma, amyotrophic lateral sclerosis, brain tumor, etc^6^. Among these underlying disorders, stroke has become one of the leading causes of death and adult disabilities. While post-stroke dysarthria (PSD) accounts for more than 20% of all dysarthria cases, relatively little research attention has been paid to this population^7^. To fill this gap, the present study was carried out to document the acoustic profile of vowel productions in patients with PSD. In particular, the temporal and spectral characteristics of six Mandarin monophthongal vowels produced by Mandarin-speaking patients with PSD were of interest in the present study.

Stroke can cause lesions at various locations in the brain. If the stroke lesions cause unilateral or bilateral damage of upper motor neurons of the pyramidal tracts, patients may suffer from unilateral upper motor neuron dysarthria or spastic dysarthria. Though defined as two different subtypes of dysarthria, unilateral upper motor neuron dysarthria or spastic dysarthria share some common speech signs such as hypernasality, imprecise consonants, slow speech rate, harsh, strangled voice, monopitch, etc. While these characteristics are based on listeners’ perceptual judgment of the dysarthric speech, an increasing number of acoustic studies have been implemented to examine the objective acoustic indicators of the articulatory abnormalities in dysarthric speech^8–10^.

Vowel is the nucleus and the most sonorant component in a syllable. Vowel production greatly contributes to the speech intelligibility. Previous studies revealed that dysarthric speech and healthy speech can be reliably distinguished by the vowel metrics derived from temporal and spectral measurements. However, different types of dysarthria in the current categorization structure did not show systematic differences on these vowel parameters^8,10^. Given the presumed homogeneity of vowel characteristics across dysarthria subtypes and the lack of acoustic studies regarding post-stroke unilateral upper motor neuron dysarthria and spastic dysarthria, the following literature review was based on vowel acoustic features of patients with various types of dysarthria. It is well known that formant patterns of vowel sounds reflect the resonance feature of different shapes of vocal tract. Formant (F1 and F2) frequency values and other related measures have long been used as indices of articulatory movement and speech motor patterns^11–15^. Dysarthric speech is characterized by distorted vowel articulation represented by deviant formant frequencies. Researchers found that patients with dysarthria usually showed centralized formant pattern which was associated with the articulatory undershoot^16–18^, unstable vowel formant patterns and reduced F2 slopes (formant transition)^8,19–23^, and abnormal F2-F1 values for both high-front vowel /i/ and high-back vowel /u/^24^. Among these F1- and F2-related metrics, F2 slope was reported to be correlated to speech intelligibility in patients with dysarthria associated with various types of diseases^9,10^. In particular, the patients with greater F2 slope tended to have higher scaled intelligibility scores. Researchers also found that F1 variability contained a significant predictive power for speech intelligibility in dysarthric speakers^25^.

In addition to the formant frequency values, another important indicator of speech intelligibility is the vowel space area. Vowel space area is defined as the size of working space surrounded by the corner vowels in a specific language. This measure has long been used to signify the accuracy of vowel articulation and the ability of speech motor control. In English, the four corner vowels /i, æ, ɑ, u/ determine the size of working vowel space as these corner vowels represent the most peripheral position the tongue can reach. Previous research showed that normal speakers produced a larger vowel space area in clear speech than in conversational speech^26,27^. In speakers with dysarthria, a substantial amount of studies have revealed that dysarthric speech, regardless of the underlying neuromuscular conditions and etiology, demonstrated reduced vowel space area^21,28–32^. The reduced vowel space area negatively impacted the speech intelligibility of dysarthric speakers^29,32,33^. For example, Weismer *et al*. (2001) compared acoustic measures in speakers with amyotrophic lateral sclerosis and Parkinson’s disease with those in healthy speakers^21^. Significantly reduced vowel space area was found in both groups of patients with neurogenic disorders relative to the healthy controls. Liu *et al*. (2005) examined the vowel space area in Mandarin-speaking young adults with cerebral palsy and found that the patients produced smaller vowel working space areas due to more centralized articulation of the corner vowels^32^.

In the temporal domain, researchers found that the articulators such as tongue, jaw, and lower lip took longer time to travel the same distance in patients with dysarthria relative to the healthy controls^4^. For example, Kent *et al*. (1979) reported prolonged speech segments and disrupted timing pattern of speech units in individuals with cerebellar disease and ataxic dysarthria^34^. Ziegler & Cramon (1986) also reported substantially elongated total word duration of the trisyllabic utterances and CV period duration in patients with spastic dysarthria after brain injury in comparison to the healthy speakers^35^. In another recent study, Rudzicz *et al*. (2011) found that vowels durations produced by dysarthric speakers were significantly longer than those by the healthy controls. In particular, the average vowel duration could be twice as long as that of the controls^36^.

Besides these conventional temporal and spectral features of vowel production, researchers also proposed a variety of additional vowel measures to quantify the degree of articulatory imprecision. Sapir *et al*. (2010) proposed an alternative acoustic measure of formant centralization ratio (FCR) to distinguish dysarthria from healthy speech^17^. The FCR was calculated using the formula: FCR = (F2u + F2a + F1i + F1u)/(F2i + F1a). The results demonstrated the effectiveness of this metric in discriminating dysarthric speech from healthy speech. Skodda *et al*. (2011) compared the vowel space area and vowel articulation index (VAI) based on the F1 and F2 of the vowels /a, i, u/ from 34 speakers with Parkinson’s disease and 32 healthy controls^37^. Adopted from the concept of Roy *et al*. (2009) and Sapir *et al*. (2010), Skodda *et al*. (2011) calculated the VAI using the formula VAI = (F2/i/ + F1/a/)/(F1/i/ + F1/u/ + F2/u/ + F2/a/)^17,37,38^. The authors found that while the reduced vowel space area only showed in the male patients with Parkinson’s disease, the VAI were significantly reduced in both male and female patients. They claimed that the VAI was superior to vowel space area in capturing the vowel articulation abnormalities. Kim *et al*. (2011) calculated mean distance between vowels and the overlap degree among vowels in addition to vowel space area and formant variability^25^. They found that these two spectral metrics showed significant correlation with the speech intelligibility in dysarthric speakers. Lansford & Liss (2014) assessed a comprehensive set of vowel metrics including FCR, mean dispersion, front dispersion, back dispersion, corner dispersion, global dispersion, and spectral overlap in addition to vowel space area and F2 slope^9^. They found that the scaled intelligibility and vowel accuracy ratings of speakers with different types of dysarthria were significantly correlated with FCR, in addition to the dynamic F2 slope and the quadrilateral vowel space area. In a recent work by Allison *et al*. (2017), the F2 interquartile range was measured in ten 5-year-old children with dysarthria caused by cerebral palsy and ten age-matched, typically developing children^28^. The results showed that the overall F2 range in children with cerebral palsy was smaller for single words with diphthongs that required large F2 transitions but not for those with monophthongs.

To date, a variety of acoustic metrics have been developed to describe vowel production deficits in dysarthria mainly for English-speaking population, and for people from a few other languages such as French, German, Japanese, and Swedish^18,39–41^. Little research has been done on the acoustic characteristics of dysarthric speech in Mandarin speakers. Mandarin differs from English on both segmental and suprasegmental levels^42^. Mandarin is a tonal language in which the four lexical tones are used to differentiate lexical meaning. Researchers found that while F0 change in Chinese is mainly associated with the laryngeal muscle tension, supralaryngeal articulatory changes may also occur during the tone production^43^. For patients with dysarthria, the laryngeal and supralaryngeal configuration for tone production may cause more difficulties in articulatory coordination for speech sounds. In addition, Mandarin has a large amount of monosyllabic words and a simpler syllable structure in comparison to English. Mandarin syllable, in the form of (C)(V)V(V)(N), has no more than four phonemes with a monophthong, diphthong or triphthong occupying the nucleus position. These phonetic features may cause distinct acoustic-phonetic characteristics of dysarthric speech in Mandarin Chinese relative to those in English. However, we lack even a basic understanding of the global profile of speech production in Mandarin speakers with dysarthria. The present study, therefore, aims to extend previous relevant studies to Mandarin-speaking population and conduct an acoustic investigation on the basic vowel features of speakers with stroke-related dysarthria. In pursuit of this research goal, the present study compared the temporal and spectral features including vowel duration, formant frequency values, vowel space area, F1 deviation, and F2 deviation between speakers with stroke-related dysarthria and healthy speakers.

## Results

### Vowel durations

**Figure 1:**
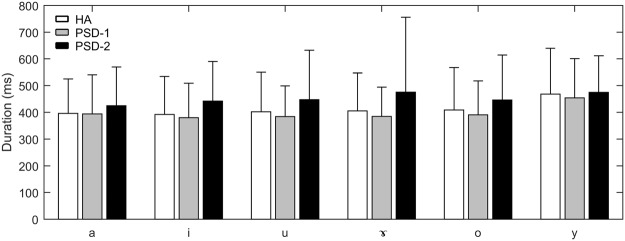
Group mean vowel durations produced by the HA, PSD-1, and PSD-2 groups for each of the six Mandarin vowels. The error bar represents 1 standard deviation (SD).

Figure 1 presents the average durations of the six Mandarin vowels for PSD-1, PSD-2, and HA groups. While the PSD-2 group showed a trend of longer durations for all six vowels than the HA group, no difference was observed between PSD-1 and HA groups on the vowel durations. One-way ANOVAs were used to compare group difference on the durations of each vowel. The results showed no significant differences among the PSD-1, PSD-2, and HA groups for any of the six vowels.

### Degree of vowel dispersion

**Figure 2:**
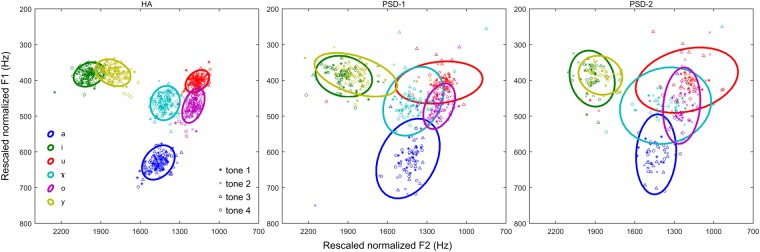
Scatter plots based on rescaled normalized midpoint F1 and F2 values of six Mandarin vowels. Data from the HA, PSD-1, and PSD-2 groups are shown on the left, middle, and right panels, respectively. Different colors and symbols represent different vowel categories. Each ellipse encompasses ~95% of data points for one vowel category.

Figure 2 shows the scatter plots of rescaled normalized midpoint F1 and F2 with each ellipse encircling ~95% of the tokens of each vowel category. The vowel tokens in the four different Mandarin tones were presented with different symbols. As shown in the left panel for the HA group, all six Mandarin vowels (i.e., /a, i, u, ɤ, o, y/) were relatively well separated in the vowel space. Within each vowel category, the vowel tokens in all four tones were clustered tightly in the F1 × F2 space. However for the PSD-1 (middle panel) and PSD-2 (right panel) groups, the vowel categories were more scattered and greatly overlapped than those in the HA group. The scattering of the back vowels /u/, /ɤ/, and /o/ was greater in the PSD-2 group than in the PSD-1 group, which indicated that the degree of vowel dispersion corresponded to the severity of dysarthria. Among these six vowels, the vowel /a/ in both dysarthric groups showed great deviation along the F1 axis. The vowel /i/ showed evident deviations in F1 and F2 for the PSD-2 group. The vowel /u/ in both PSD-1 and PSD-2 groups showed remarkable deviations along the F2 axis while the vowel /o/ showed more deviations in F1 than in F2 for both groups of dysarthric speakers. For the vowel /ɤ/, the PSD-1 speakers showed greater deviation in F1 than in F2 while the PSD-2 speakers showed greater deviations in both F1 and F2 relative to the HA speakers. Note that the vowel tokens in the four tones did not show distinct distributional patterns within each vowel category. However, for both groups of patients with dysarthria, greater deviations could be observed for tone 3 in certain vowels. Due to the small numbers of tokens for each vowel at each tone, no quantitative assessment of the potential effects of lexical tones on the vowel acoustics was performed. In the following analyses, data on vowels of all four tones were pooled together and the potential effects of lexical tones on the vowel acoustics were not considered.

### Sizes of vowel ellipse areas

**Figure 3:**
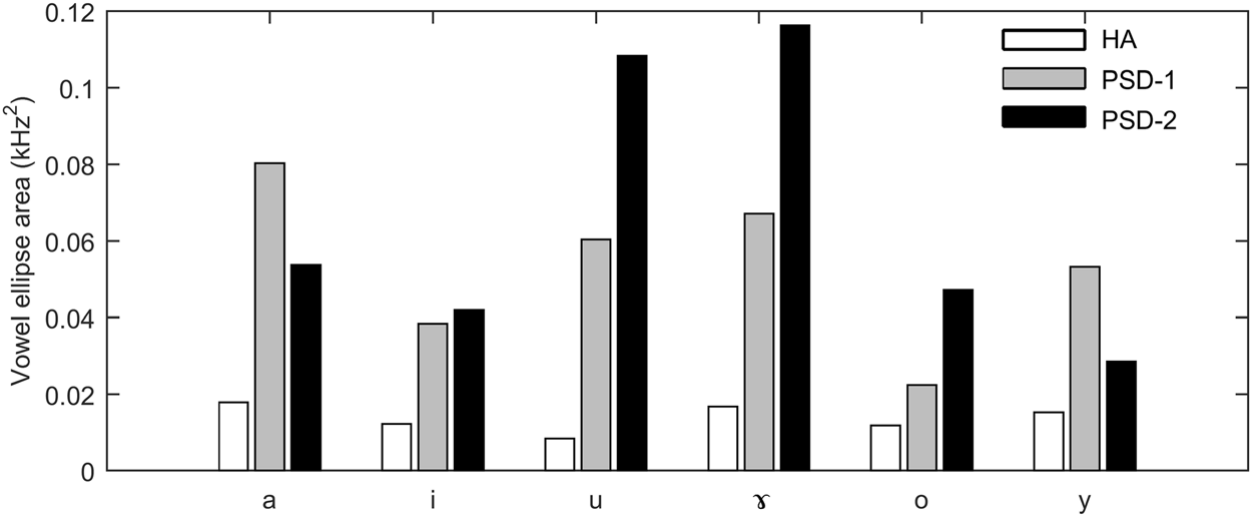
Vowel ellipse areas for individual Mandarin vowel categories in the HA, PSD-1, and PSD-2 groups.

Figure 3 presents the size of vowel ellipse area for each vowel in each group of speakers. Not surprisingly, the vowel ellipses of all six vowels in the two PSD groups were substantially larger than those in the HA group. In particular, the PSD-2 speakers produced the vowels /u, ɤ, o/ with larger ellipse areas than the HA and PSD-1 speakers while the PDS-1 speakers produced the vowels /a, y/ with larger ellipse areas than the HA and PSD-2 speakers. Among the six vowels, both groups of patients with PSD produced greater vowel ellipses areas for the vowels /u, ɤ, a/ than for the vowels /i, y, o/. These results showed that the speakers with PSD produced these vowels with great positional deviations from the HA targets.

### Vowel space area

**Figure 4:**
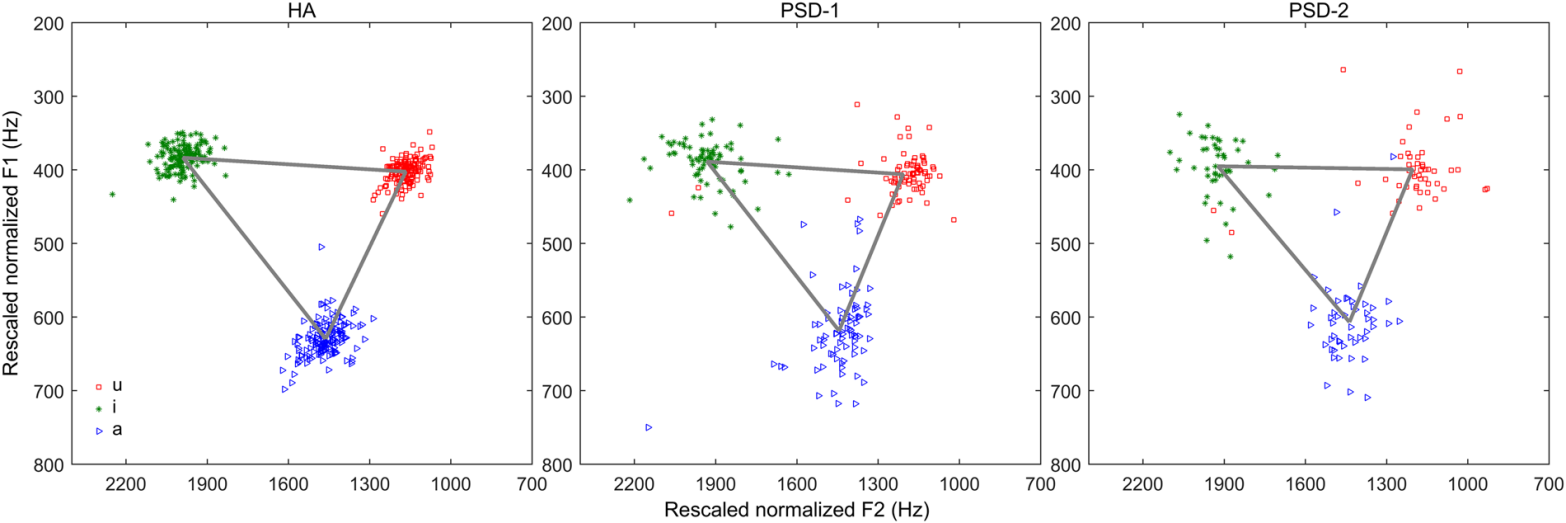
Mandarin vowel space of the HA (left), PSD-1 (middle), and PSD-2 (right) groups. Each data point represents the rescaled normalized F1 and F2 values of /a, i, u/. The triangle was formed based on the group mean data of the rescaled normalized F1 and F2 values of /a, i, u/.

**Figure 5:**
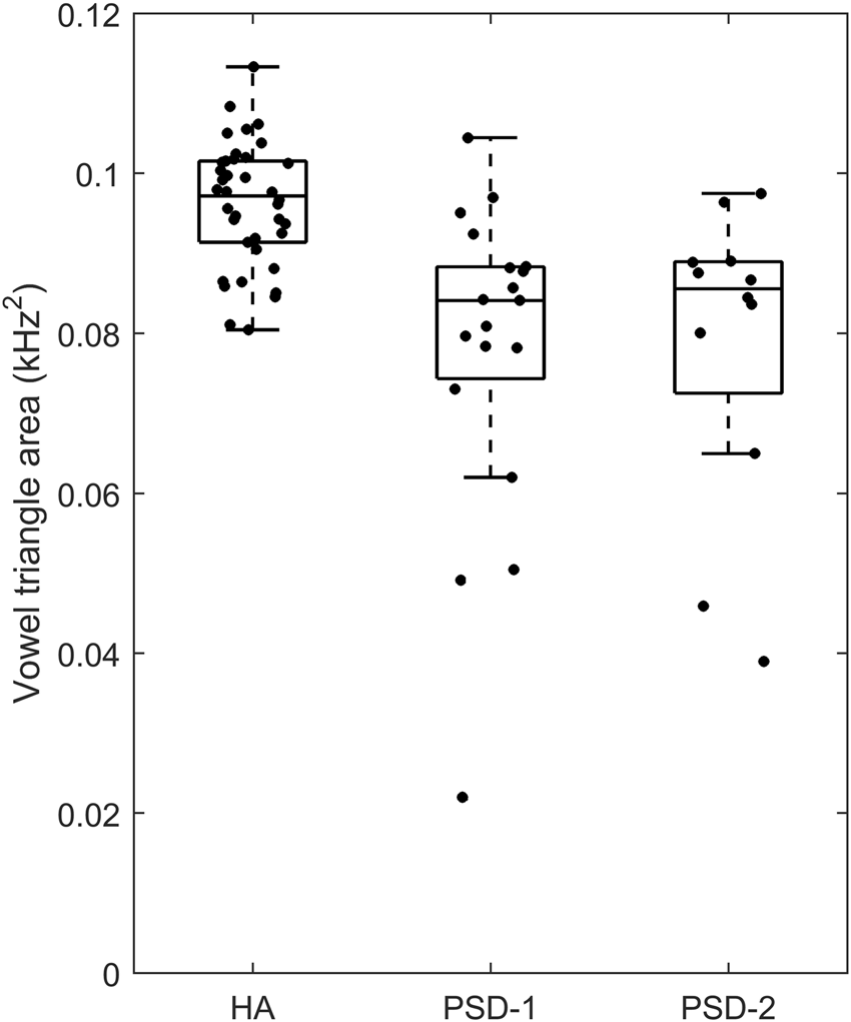
Boxplots of vowel space areas of the HA, PSD-1, and PSD-2 groups. Each data point represents vowel space from one participant. Each box shows horizontal lines at the lower quartile, median, and upper quartile values. The whiskers show the range of the data and the data points out of the whiskers are outliers. Some jitters along the abscissa were applied to the data for better visual representation.

Figure 4 shows the dispersions of the three Mandarin corner vowels in F1 × F2 space. The area formed by the triangle defined the vowel space area. Figure 5 shows the vowel space areas of individual speakers for the three groups. The group average vowel space area was 0.096, 0.079, and 0.075 kHz^2^ for the HA, PSD-1, and PSD-2 groups, respectively. A one-way ANOVA conducted on the vowel space area yielded a significant difference among the HA, PSD-1, and PSD-2 groups [F (2, 66) = 13.81, p < 0.001]. Post hoc analysis showed a smaller vowel space in both PSD-1 and PSD-2 than in the HA group. However, no group difference was found between the PSD-1 and PSD-2 groups.

### Formant deviation

**Figure 6:**
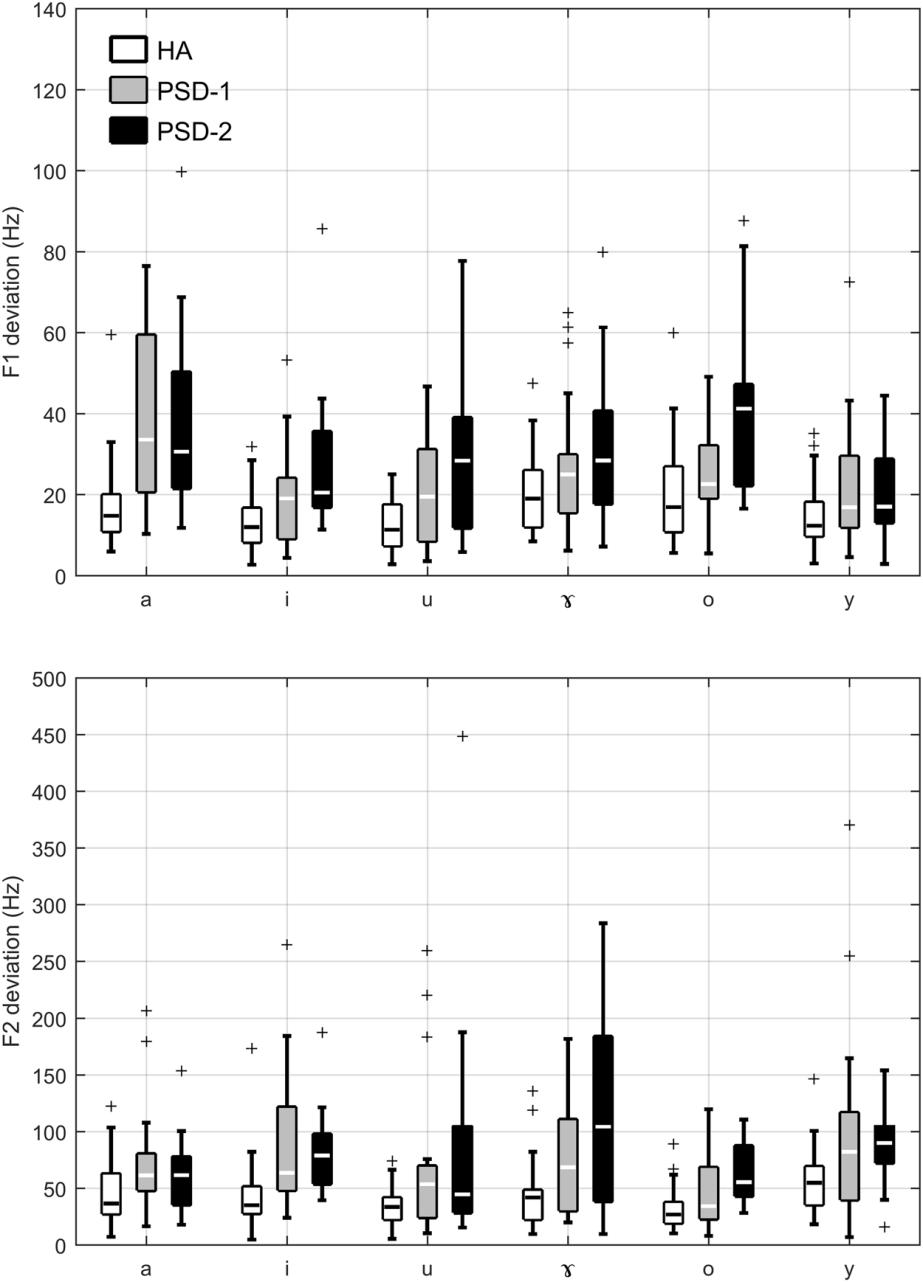
Boxplots showing the F1 deviation (top panel) and F2 deviation (bottom panel) of the HA, PSD-1, and PSD-2 groups. Each box shows horizontal lines at the lower quartile, median, and upper quartile values. The whiskers show the range of the data and the data points out of the whiskers are outliers.

Figure 6 shows the F1 and F2 deviations of individual healthy speakers or patients with PSD relative to the healthy target for all six vowels. Generally speaking, F1 varies with the change of tongue height and F2 varies with the change of tongue advancement. Therefore, the F1 and F2 deviations can be used to index the degree of articulatory imprecision for patients with PSD in tongue height and tongue advancement. Both groups of patients showed greater deviations on both F1 and F2 for most vowels in comparison to the healthy speakers. Between these two groups of patients, the PSD-2 patients who were categorized as the moderate-to-severe group on the basis of speech accuracy scores showed greater formant deviations in F1 and/or F2 for the vowels /i, u, ɤ, o/ than the PSD-1 patients who were categorized as the mild group. For some other vowels, such as /a/ and /y/, the PSD-1 patients showed similar formant deviations to those seen in the PSD-2 patients. It is also noteworthy that both groups of patients showed greater variability than the healthy speakers. As for the F1 deviations, among the six vowels, both groups of patients showed greater deviations for the vowels /a/, /u/, /o/, and /ɤ/ than the vowels /i/ and /y/. As for the F2 deviations, while the PSD-1 patients showed greater deviations for the vowels /i/, /ɤ/, and /y/ than for the other vowels, the PSD-2 patients showed greater deviations for the vowels /u/ and /ɤ/ than for the other vowels.

A one-way MANOVA test was conducted to compare the difference among the three groups of speakers on the formant deviation for each vowel. Significant multivariate effects were found for all six vowels, /a/: Wilk’s Lambda = 0.700, F (4, 130) = 6.353, p < 0.0001; /i/: Wilk’s Lambda = 0.676, F (4, 130) = 7.033, p < 0.0001; /u/: Wilk’s Lambda = 0.750, F (4, 130) = 5.032, p = 0.001; /ɤ/: Wilk’s Lambda = 0.725, F (4, 130) = 5.666, p < 0.0001; /o/: Wilk’s Lambda = 0.653, F (4, 130) = 7.719, p < 0.0001; /y/: Wilk’s Lambda = 0.846, F (4, 130) = 2.839, p = 0.015. Subsequent univariate tests with Bonferroni post-hoc tests (adjusted for multiple comparisons) were conducted for F1 and F2 deviations respectively for individual vowels. The statistical results were summarized in Table 1. These results indicated that the patients in the PSD-2 group showed deficiencies in both directions of tongue placement in their vowel productions.

**Table 1:** Summary of univariate test and Bonferroni post hoc tests for group difference on F1 deviation and F2 deviation.

Vowel	Formant	Univariate test		Bonferroni post hoc
		F	p	pairwise comparison
a	F1	F(2, 66) = 13.670	<0.0001	HA < PSD-1, HA < PSD-2
	F2	F(2, 66) = 3.698	0.030	HA < PSD-1
i	F1	F(2, 66) = 8.398	0.001	HA < PSD-2
	F2	F(2, 66) = 9.718	<0.0001	HA < PSD-1, HA < PSD-2
u	F1	F(2, 66) = 10.356	<0.0001	HA < PSD-2
	F2	F(2, 66) = 4.838	0.011	HA < PSD-2
ɤ	F1	F(2, 66) = 4.487	0.015	HA < PSD-2
	F2	F(2, 66) = 11.235	<0.0001	HA < PSD-2,
o	F1	F(2, 66) = 11.809	<0.0001	HA < PSD-2, PSD-1 < PSD-2
	F2	F(2, 66) = 9.775	<0.0001	HA < PSD-2,
y	F1	F(2, 66) = 3.127	>0.050	
	F2	F(2, 66) = 4.139	0.020	HA < PSD-1

## Discussion

The primary goal of the present study was to investigate the acoustic characteristics of vowel production in Mandarin-speaking patients with PSD. With detailed acoustical analyses, we found that certain aspects of the vowel acoustic features in Mandarin-speaking patients with PSD, mainly represented in the spectral features, showed distinctive patterns from the similarly-aged healthy adults.

Many early studies reported that English-speaking patients with dysarthria showed reduced tongue, jaw, and lower lip movements as compared to the healthy speakers^3^. The reduced articulatory movement was manifested acoustically in reduced vowel space area and centralized vowels in the F1 × F2 acoustic space. In the present study, we also found reduced vowel space area in Mandarin-speaking people with PSD. This result is similar to the findings reported in previous studies in English-speaking individuals^21,32,44^. Researchers have proposed that the size of vowel space area represents gross motor control ability of articulators, such as tongue, jaw, lip, and velar coordination^45,46^. In addition, the size of vowel space has a positive relationship with speakers’ intelligibility. In both normal individuals and speakers with speech motor disorders, those with a larger vowel space area were judged to be more intelligible^27,29,47^. In the present study, we observed that both groups of patients with PSD had smaller vowel space areas than the HA controls. However, the PSD-2 speakers who were perceived with less articulation accuracy did not always produce smaller vowel space areas than the PSD-1 speakers who were perceived with only mild articulatory problems. This might be because the intelligibility rating scores used for the categorization of PSD subgroups were not solely based on the vowel productions but rather based on the entire phonetic repertoire including all speech segments. It is possible that certain patients with PSD who were categorized in the moderate-to-severe (PSD-2) group did not show remarkable deficits in vowel articulation. Therefore, they did not always show smaller vowel space areas than the PSD-1 patients.

It is known that reduced vowel space is usually caused by centralized vowels, in particular, corner vowels. In the present study, both groups of patients with PSD showed more reduced group average vowel space than the healthy adults. However, instead of ubiquitously showing centralized corner vowels, both groups of patients with PSD produced certain corner vowels with more peripheral articulatory positions than the healthy controls. The more peripheral locations and more scattered vowel tokens in the dysarthric speakers might suggest that the patients with PSD showed off-target articulatory gestures which were reflected as greater variability of vowel productions in the acoustic space. In addition to the corner vowels, the patients with PSD also demonstrated different features on the other vowels /y, o, ɤ/ when compared to the HA speakers. As shown in Fig. 2 for vowel dispersion and ellipses, both groups of patients produced widely scattered non-corner vowels /y, o, ɤ/ that were highly overlapped with other vowels. In particular, the vowel /y/ was almost completely merged with the vowel /i/ in both PSD-1 and PSD-2 speakers. The vowel /ɤ/ was also greatly overlapped with the vowel /u/ in both groups of PSD patients. In the PSD-2 speakers, the vowel /o/ was entirely overlapped with the vowel /ɤ/. A previous study found that the degree of spectral deviation and overlap among vowels impacted speech intelligibility in English-speaking patients with dysarthria^25^. In the present study, the two groups of PSD speakers especially PSD-2 speakers who showed substantial overlaps in the vowel space were also judged with much poorer overall speech intelligibility than the healthy speakers.

A close comparison of vowel dispersions and ellipses areas revealed that the patients with PSD seemed to show greater dispersions in the back vowels than in the front vowels (Figs 2 and 3). As stated earlier, the acoustic characteristics of vowel formants reflect the articulatory position of tongue placement. To better quantify the degree of imprecision of tongue placement; we calculated the formant deviations of individual vowel tokens for all participants relative to the healthy targets. The results suggested that the patients with PSD showed greater deviations than the healthy adults in both F1 and F2 for all six vowels (Fig. 6). Specifically, PSD-1 speakers showed greater deviations for the vowels /a/ and /ɤ/ in F1 and the vowels /y/ and /u/ in F2 than the healthy controls. PSD-2 speakers showed greater deviations for the vowels /a/, /i/, and /o/ in F1 and the vowels /u/ and /ɤ/ in both F1 and F2 than the healthy controls. These results suggested that the patients did not accurately form the articulatory gestures for vowel production in both tongue height and tongue advancement.

Unlike the distinctive patterns of spectral features of vowel productions, the vowel durations in the PSD speakers did not show significant differences from the HA controls. Several previous studies used auditory-perceptual ratings to examine the speech characteristics of patients with dysarthria associated with stroke at various lesion locations^48–53^. One of the speech features examined in these studies was the speech rate and durations of syllables. These studies reported that patients with stroke-related dysarthria were characterized by reduced rate of speech and prolonged syllables. Bunton & Weismer^54^ compared the acoustic features including formant values, F0 and vowel duration of nine pairs of high-low vowels embedded in CVC structures between patients with dysarthria caused by Parkinson’s disease, amyotrophic lateral sclerosis, or cerebrovascular accident and age-matched healthy adults. The authors found that the patients with dysarthria showed a pattern of longer durations for many vowel pairs relative to the health adults. However, none of the differences were statistically significant^54^. Similar to Bunton & Weismer^54^ study, the present study revealed a tendency of longer vowel duration in PSD-2 speakers relative to the HA controls and the PSD-1 speakers, but no statistically significant result was yielded. Unlike English language that has a large number of multisyllabic words, a majority of Mandarin words are monosyllabic or disyllabic words. In the present study, the six vowels were embedded in monosyllabic CV words. Presumably, the Mandarin CV syllables are easy to produce and involve less articulatory coordination, which might partially explain the lack of statistical significance in vowel duration between the patients with dysarthria and healthy adults in the present study.

Taken together, the present study described the detailed acoustic-phonetic features of vowel production in Mandarin-speaking patients with PSD. We found that the patients with PSD differed from the HA controls mainly in spectral features represented in the relative position in the F1 × F2 space, vowel space area, and F1 and F2 deviations. These acoustic differences between the PSD and HA speakers indicate that the patients with stroke-related dysarthria tend to form imprecise and more diverse articulatory configurations in comparison to healthy adults. These findings expand our understanding of the vowel acoustic features of this population for clinic speech assessment and treatment design. For example, speech therapists should emphasize the articulatory training for the non-corner vowels as these vowels demonstrated greater deviations and variations relative to the corner vowels in the patients with PSD. Furthermore, for those vowels that showed great acoustic overlaps in the acoustic vowel space, clinicians should develop training activities to help them differentiate the overlapped vowel pairs. Since the current study focused on the midpoint formant frequency values, it remains unknown whether speakers with dysarthria will show different patterns from HA speakers on vowel dynamic spectral changes. Mandarin has a rich number of diphthongs and triphthongs that include two or more vowel targets in the same syllable and require more delicate articulatory controls^55^. It is reasonable to assume that patients with PSD may show more difficulties in producing these compound vowels and show different formant dynamics from the HA speakers. Moreover, the present study focused on vowel productions in monosyllables. Future studies on consonant features and speech segments in connected speech as well as spontaneous speech are necessary to establish a global profile of acoustic characteristics in dysarthric speakers.

As a preliminary study examining the vowel acoustic features in Mandarin-speaking adults with dysarthria, the current study presented several other limitations. First, Mandarin is a tonal language. It remains unknown whether the laryngeal control for tone production shows deficiency in Mandarin-speaking patients with dysarthria and whether this factor interferes with the supralaryngeal configuration for vowel production. Previous studies on tone-vowel interactions have revealed some small but consistent effects in normal speakers, challenging the idea of independent control of tone (source) and vowel (filter) production units^56–58^. However, no data is available on the tone-vowel interactions in dysarthric speakers. The present study pooled vowel data across different lexical tones due to a limited number of speech tokens at each tone, which made it infeasible to examine the potential effects of lexical tones on vowel formant features. Indeed, we observed that the patients with PSD tended to produce certain vowels in tone 3 with more deviated position in the F1 × F2 space. A future study examining the accuracy of tone production and interactions between tone and vowel production should be implemented. In addition, the present study only included two naive listeners to evaluate the speech intelligibility. Although naive listeners represent the conversational partners the patients likely to encounter in their daily life, they are less likely to conduct analytical perceptual evaluations to catch the subtle deviations and deficits presented in dysarthric speech. In future studies, more listeners including professionally-trained listeners should be recruited to ensure more reliable intelligibility rating scores.

## Conclusion

This study documented the acoustic features of vowel production in Mandarin-speaking patients with post-stroke dysarthria. The results showed that all vowel categories in the patients with PSD were produced with more variability than in the healthy speakers. Great overlaps between vowel categories and reduced vowel space were observed in the patients. The magnitude of the vowel dispersion and overlap between vowel categories increased as a function of the severity of the disorder. The deviations of the vowel acoustic features in the patients from the healthy speakers may provide guidance for clinical rehabilitation to improve the speech intelligibility of patients with PSD.

## Methods

### Participants

The participants included 31 native Mandarin-speaking patients (19 males and 12 females) with post-stroke dysarthria. The age of the dysarthric speakers ranged from 25 to 83 years old [mean ± standard deviation (SD): 56.74 ± 16.40 years]. All participants presented with a slow speech rate and strained-strangled vocal quality although other dysarthric characteristics varied among the participants. All participants went through physical examination, Frenchay dysarthria assessment, and other auxiliary examinations (such as brain CT, MRI, and direct/indirect laryngoscopy). The detailed demographic information and medical information related to stroke are provided in Table 2. Before the stroke occurred, all patients had no speech-related impairments and were able to communicate fluently in Mandarin. They had no alexia, visual, or severe auditory comprehension impairments, and had pure-tone thresholds at 500, 1000, and 2000 Hz of ≤25 dB HL in at least one ear. The patients had received no systematic speech-language therapy prior to the participation in the present study although some patients received informal speech practice occasionally. Any patients who had no articulation at all or had only nonsense vocalization were excluded.

**Table 2:** Demographic information, radiological results, and intelligibility scores for all participants.

Subject Number	Sex	Age	Diagnosis	Lesion side	Lesion sites	Intelligibility (%)	Group Assignment
1	M	47	Hemorrhagic Infarction	L	BG, FL, TL, PL, IL	76.52	1
2	M	43	Cerebral Hemorrhage	L	BG, TL	57.62	2
3	M	50	Cerebral Infarction	L	BG, FL, TL, PL, OL	47.87	1
4	M	42	Cerebral Infarction	L	BG	94.21	2
5	M	71	Cerebral Infarction	L, R	CS, FL, PL, OL	90.24	2
6	M	35	Cerebral Hemorrhage	L	BG, THA, CR	91.77	2
7	M	78	Cerebral Infarction	R	BG, CR, CS	81.1	2
8	M	25	Cerebral Infarction	L, R	FL, PL	97.56	2
9	M	54	Cerebral Infarction	L	FL, PL, TL	38.11	1
10	M	28	Cerebral Hemorrhage	R	BG	96.04	1
11	M	68	Cerebral Infarction	L	PL, OL	86.59	2
12	M	42	Cerebral Infarction	R	PL, TL	61.89	1
13	M	57	Cerebral Infarction	L, R	BG, CR, FL	89.94	1
14	M	50	Cerebral Infarction	R	PL, TL, OL	81.1	2
15	M	75	Cerebral Infarction	L, R	CS	82.93	2
16	M	76	Cerebral Hemorrhage	L, R	CR, OL	96.34	1
17	M	55	Cerebral Infarction	L, R	BG, CC, PL	79.88	1
18	M	67	Cerebral Infarction	L, R	BG, CR, CS	53.05	1
19	M	68	Cerebral Infarction	R	BG, CN, CS, FL, TL,PL	82.93	1
20	F	82	Cerebral Infarction	L, R	BG, THA, FL	78.96	1
21	F	73	Cerebral Infarction	L, R	BG, CR, CS	12.8	2
22	F	27	Cerebral Hemorrhage	R	BG, TL	89.33	1
23	F	52	Cerebral Hemorrhage	R	BG, TL, PL	74.09	1
24	F	69	Cerebral Infarction	L	BG, FL, TL	67.99	2
25	F	56	Cerebral Hemorrhage	L	BG, CS, FL	70.43	1
26	F	83	Cerebral Infarction	L, R	BG, THA, CR	58.54	1
27	F	66	Cerebral Infarction	L, R	CS, CR	63.11	1
28	F	59	Cerebral Infarction	L, R	CS, FL, PL	97.87	1
29	F	71	Cerebral Infarction	L	THA, PL, TL, OL, SCC	96.65	1
30	F	43	Cerebral Infarction	L, R	BG	55.49	2
31	F	47	Cerebral Hemorrhage	L	BG, CR, CS	93.29	1

BG - Basal ganglia; CC - Corpus callosum; CN - Caudate nucleus; CR - Corona radiata; CS - Centrum semiovale; FL - Frontal lobe; IL - Insular lobe; OL - Occipital lobe; PL - Parietal lobe; SCC - Splenium of corpus callosum; THA - Thalamus; TL - Temporal lobe.

The control group included 38 healthy adults (HA) (19 males and 19 females) in a similar age range (21 to 76 years old; mean ± SD: 45.89 ± 13.02 years). Some participants in the HA group were the family members of the dysarthria groups. The healthy adults had pure-tone thresholds at 500, 1000, and 2000 Hz of ≤25 dB HL in at least one ear with no reported hearing or speech disorders. The use of human subjects was reviewed and approved by the Institutional Review Board of Jinan University School of Medicine. All research was performed in accordance with relevant guidelines and regulations. Informed consent was obtained from all participants.

### Speech materials and data collection

Each participant produced a list of 24 Mandarin monosyllables that contained six Mandarin vowels /a, i, u, ɤ, o, y/. These vowels are traditionally regarded as the six monophthongal vowel phonemes in Mandarin although some recent studies suggested that the vowel /o/ is phonetically realized as a diphthong /uo/^59,60^. The word list included 6 syllables (‘ba’, ‘bi’, ‘du’, ‘ge’, ‘bo’, ‘yu’) in all four Mandarin tones, resulting in 24 Mandarin Chinese words. The 6 syllables have simple structures with stops or no consonants in the syllable-initial position, which are presumably easy to produce for dysarthric speakers especially the patients with severe problems in articulatory gesture formation and coordination. During the recording session, each participant was seated in a sound-proof room. The experimenter articulated each syllable in sequence and then each participant was required to repeat the same syllable in all four tones. The experimenter did not make corrections to the participants during recording, but participants’ self-corrections were allowed. All speech recordings were conducted by the same experimenter. All speech samples were recorded through a Sony portable digital recorder (Model ZOOM H4N) with a 44.1-kHz sampling rate and a 16-bit quantization rate. For each participant, the first valid sample of each word in each tone was used for the acoustic analysis. By excluding the missing tokens and the tokens with insufficient acoustic energy, a total of 731 out of 744 tokens from the PSD groups and a total of 912 tokens from the HA group were used for the acoustic analyses.

### Categorization of dysarthric speakers

Prior to further acoustic analyses, the speakers with dysarthria were classified into different groups based on listeners’ rating on the accuracy of their production of a list of 82 Mandarin monosyllabic words that covers the entire phonetic repertoire and Mandarin lexical tones. This word list provides a good assessment of the overall speech intelligibility in speakers with dysarthria. The 24 words used for acoustic analyses in the present study were included in this word list. Two normal-hearing, native Mandarin-speaking adult listeners (a male and a female) were recruited for the perception task. The two listeners had no more than incidental experience with persons having speech-language disorders. At the beginning of the perceptual task, the listeners were informed that they were going to listen to real words in Mandarin produced by individuals with speech disorders. Then, the listeners were provided with the target words and were asked to rate to what extent the stimuli they heard matched the target words. Following the three-point rating scale for word accuracy in Kim *et al*.^25^, the listeners rated the stimuli as ‘0’ (the target word being perceived as other word or nonsense pronunciation), ‘1’ (approximated the target word with blurred pronunciation), or ‘2’ (accurate pronunciation). Each listener was allowed to listen to each stimulus several times as needed (usually no more than 3 times). After all tokens had been rated, an inter-rater consistency was checked. Any discrepancy in rating was re-evaluated and reconciled with perfect agreement between the two listeners. Note that the perceptual rating was based on the overall intelligibility which included the vowel, consonant, and tone production. The score reflected each patient’s accuracy for the whole word rather than just for the vowel production. As controls, two other normal-hearing, native Mandarin-speaking adult listeners were recruited for the intelligibility task on the speech tokens of all HA speakers. The mean intelligibility score of the HA speakers was 97.85% correct (SD = 2.85%).

Given that there are no widely-accepted criteria to distinguish the degree of severity for different types of dysarthria, a K-means clustering approach was used to categorize the dysarthric speakers on the basis of the accuracy scores of individual dysarthric speakers. In the K-means clustering analysis, the sum of all intelligibility scores for individual participants with PSD (N = 31) was fed into the K-means algorithm to partition the intelligibility scores into three categories which corresponded to mild, moderate, and severe groups. This partition minimized the sum, over all 3 clusters, of the within-cluster sums of point-to-cluster-centroid distances. The categorization result showed that the severe group had only two patients with PSD with the two lowest accuracy scores. Thus, these two most severe patients were grouped with the patients who had the accuracy rating scores in the moderate level. Therefore, the patients with dysarthria were divided into two subgroups: PSD-1 (mild, n = 19, group mean intelligibility ± standard deviation: 88.6% ± 7.1% correct) and PSD-2 (moderate-to-severe, n = 12, group mean intelligibility ± standard deviation: 55.1% ± 16.6% correct). Information regarding the group assignment for each patient is provided in Table 2.

### Data analysis

These recorded materials were segmented into separate syllables and saved as individual wave files using CoolEdit 2000 (Syntrillium Software, Scottsdale, AZ). The spectrographic program TF32 (Milenkovic, 2003) was used to determine the frequencies of the first two formants, F1 and F2, at the midpoint location over the course of vowel duration in each token. The formant extraction was based on linear predictive coding analysis and the extracted formant tracks were displayed on the spectrogram. Manual correction was performed when errors of automatic extraction were found. The onset and offset of each vowel were determined through a visual check of the waveform and the spectrogram. Vowel onset was set at the zero-crossing point of the first period with visible formant track. Vowel offset was marked at a point representing the cessation of periodicity with visible formant track.

To eliminate variation of formant frequency values caused by physiological differences (e.g., vocal tract sizes associated with gender and age) in speakers, all formant frequency values were normalized using the Lobanov (1971) method^61^. Lobanov’s method is one of the vowel-extrinsic normalization approaches. It was recommended as one the most effective normalization approach for vowel acoustic studies^62^. The Lobanov normalization was based on z scores of each formant value:

1 $${{F}}_{{n}}{[{V}]}^{{N}}=({{F}}_{{n}}[{V}]-{MEA}{{N}}_{{n}})/{S}{{D}}_{{n}}$$

where *F*_*n*_[*V*]^*N*^ is the normalized value for *F*_*n*_[*V*] (i.e., for formant *n* of vowel *V*), and *MEAN*_*n*_ and *SD*_*n*_ are the mean value and standard deviation for formant *n* for a particular speaker.

All normalized z-scores were then rescaled to Hz-like values to facilitate further interpretation via a process using the following formulas proposed by Thomas and Kendall^63^:

2 $${\rm{F}}^{\prime} 1=250+500\,({{\rm{F}}}_{1}^{{\rm{N}}}-{{\rm{F}}}_{1}^{{\rm{N}}}MIN)/({{\rm{F}}}_{1}^{{\rm{N}}}MAX-{{\rm{F}}}_{1}^{{\rm{N}}}MIN)$$

3 $${\rm{F}}\text{'}2=850+1400\,({{\rm{F}}}_{2}^{{\rm{N}}}-{{\rm{F}}}_{2}^{{\rm{N}}}MIN)/({{\rm{F}}}_{2}^{{\rm{N}}}MAX-{{\rm{F}}}_{2}^{{\rm{N}}}MIN)$$

where ${{\rm{F}}}_{1}^{{\rm{N}}}$ and ${{\rm{F}}}_{2}^{{\rm{N}}}\,\,$are the normalized *z* scores for F1 and F2, respectively. *MAX* and *MIN* denote the maximum and minimum values across all normalized *z* scores.

To quantify the degree of dispersion of each vowel category in the vowel space for the PSD and HA participants, a vowel ellipse^64^ for each vowel category in each group of speakers was plotted based on the midpoint formant values. First, the rescaled normalized F1 × F2 scatter plot for each vowel was fitted linearly and the positive angle of the linear fit was taken as the direction of the semimajor axis of the ellipse. The ellipse center was determined by the mean of the rescaled normalized F2 values along the fitting line. A perpendicular line to the fitting line defined the direction of the semiminor axis. The lengths of the semimajor and the semiminor axes were determined by two standard deviations of all F1 and F2 data points along the respective lines. The ellipse space areas of individual vowels in the PSD-1, PSD-2, and HA groups were calculated using the formula:

4 $${\rm{Ellipse}}\,{\rm{space}}\,{\rm{area}}=a\times b\times \pi $$

where *a* and *b* are the semimajor and semiminor, respectively. Vowel space for Mandarin Chinese is defined by the triangle formed by the F1 and F2 of the three corner vowels /a, i, u/. The area was calculated using the following formula:

5 $${\rm{Vowel}}\,{\rm{triangle}}\,{\rm{area}}={\rm{ABS}}\{[{\rm{F}}1{\rm{a}}\times ({\rm{F}}2{\rm{u}}-{\rm{F}}2{\rm{i}})+{\rm{F}}1{\rm{i}}\times ({\rm{F}}2{\rm{a}}-{\rm{F}}2{\rm{u}})+{\rm{F}}1{\rm{u}}\times ({\rm{F}}2{\rm{i}}-{\rm{F}}2{\rm{a}})]/2\}$$

where ABS is the absolute value. F1a is the F1 value of vowel /a/, F2u is the F2 value of vowel /u/, and so on and so forth.

As speakers with dysarthria commonly show difficulties in tongue placement for precise vowel production, to better quantify the deficits of tongue movement along the direction of tongue height or advancement, F1 deviation and F2 deviation were calculated respectively. For each formant of each vowel, the group mean of healthy adults was obtained and served as the healthy target. The absolute difference between each HA or PSD participant and the healthy target was calculated for each formant.

The vowel acoustic measures were subject to statistical analyses. Considering that we had a small number of participants and each participant produced each vowel in each tone only once, the acoustic measures for individual vowels were collapsed across four tones. As the effect of vowel quality on the vowel acoustic features was expected, which was not of interest in the present study, one-way ANOVA tests were implemented on vowel duration and vowel space area, to determine if the three groups of speakers (one HA group and two PSD groups) showed any differences on these vowel metrics for each vowel. For the formant deviation, a MANOVA test was conducted for each vowel with the F1 deviation and F2 deviation as the dependent variables and the speaker group as the independent variable.
